# Two new species of the genus *Trouessartia* (Acari, Trouessartiidae) from laughingthrushes (Passeriformes, Leiothrichidae)

**DOI:** 10.3897/zookeys.571.7724

**Published:** 2016-03-07

**Authors:** Ioana Cristina Constantinescu, Ioana Cobzaru, D. Khlur B. Mukhim, Costică Adam

**Affiliations:** 1 “Grigore Antipa” National Museum of Natural History, Sos. Kiseleff no.1, 011341 Bucharest, Romania; 2 Ecology, Taxonomy and Nature Conservation Department, Institute of Biology, Romanian Academy, Splaiul Independenței no. 296, 060031 Bucharest, Romania; 3 Zoology Department, Lady Keane College, 793001 Shillong, Meghalaya, India

**Keywords:** Acari, Trouessartiidae, new species, systematics

## Abstract

Two new feather mite species of the genus *Trouessartia* Canestrini are described from laughingthrushes (Passeriformes: Leiothrichidae) captured in Meghalaya (India): *Trouessartia cyanouropterae* **sp. n.** from *Actinodura cyanouroptera* (Hodgson) and *Trouessartia alcippeae* **sp. n.** from *Alcippe nipalensis* (Hodgson). It is the first time when species of the genus *Trouessartia* are described from leiothrichids.

## Introduction

The feather mite genus *Trouessartia* Canestrini, 1899 comprises 110 species associated predominantly with birds from the order Passeriformes. A revision of this genus including 71 species was performed by Santana (1976); other species were described in subsequent fourty year by various authors (Mauri and De Alzuet 1968, Černý and Lukoschus 1975, Gaud 1977, Černý 1979, Mironov 1983, Gaud and Atyeo 1986, 1987, Mironov and Kopij 1996, 2000, Mironov and Galloway 2002, OConnor et al. 2005, Carleton and Proctor 2010, Constantinescu et al. 2013, Mironov and González-Acuña 2013, Hernandes 2014, Hernandes and Valim 2015). Santana (1976) estimated that species redescribed in his revision represent 10–15% of the real number of species. Hernandes and Valim (2015) suggested that this genus could possibly include over 500 species. Barreto et al. (2012) found 22 undetermined species of *Trouessartia* from Colombia, Silva et al. (2015) found 15 undetermined species of *Trouessartia* from Brazil and Atyeo (in McClure and Ratanaworabhan 1973) reported 162 undetermined species of the genus from Asia (unfortunately new species from this material have never been described).

In this paper two *Trouessartia* species are described from passerine birds of family Leiothrichidae, from the hosts *Actinodura cyanouroptera* (Hodgson) and *Alcippe nipalensis* (Hodgson). Atyeo (in McClure and Ratanaworabhan 1973) mentioned the existence of two new species of *Trouessartia* on these bird hosts, but as we noted above, that material remained undescribed. Thus, it is the first time when species of the genus *Trouessartia* are described from leiothrichid birds. Santana (1976) arranged some of species into five species groups, which were rather summarily defined. Gaud and Atyeo (1986, 1987) revised and gave expanded characteristics to the *appendiculata* and *minutipes* species groups; Mironv and Kopij (2000) revised *tenuipilata* species groups and established two more groups, *viduae* and *africana*. Both new species described herein cannot be referred to any of seven species groups that have been previously established in the genus *Trouessartia*, because of having a distinct combination of characters.

## Materials and methods

The material used in the present paper was collected in Meghalaya (India) in February 2013. The birds were captured using mist-nets, identified, visually checked for the presence of mites and after collecting them were released back to the wild. Mite specimens were taken from birds manually with a needle and placed in vials with ethanol 96%. Later, in the laboratory, the mite specimens were cleared in lactic acid and mounted on microscope slides in Hoyer’s medium. Drawings were made using an Olympus CX21 microscope, with a camera lucida drawing device. The bird specimens were identified according to Rasmussen and Anderton (2012) and Grimmett et al. (2011), and the taxonomy of the birds follows Clements et al. (2015). The setation of mite’s body follows that of Griffiths et al. (1990) with modifications of Norton (1998) concerning coxal setae, while the setation of legs follows Gaud and Atyeo (1996). Descriptions of *Trouessartia* species are given according to the standards proposed for mites of the genus *Trouessartia* and related genera (Orwig 1968, Santana 1976), and the measuring techniques of particular structures used in the present paper were described by Mironov and González-Acuña (2013). We give the full set of measurements for a holotype (male) and a range of measurements for corresponding paratypes. All measurements are in micrometres (μm). The holotypes and all paratypes of the new species are deposited in the Acarological Collection of the “Grigore Antipa” National Museum of Natural History, Bucharest, Romania.

## Results

### Family Trouessartiidae Gaud, 1957 Genus *Trouessartia* Canestrini, 1899

#### Trouessartia cyanouropterae

Taxon classificationAnimaliaPasseriformesTrouessartiidae

Constantinescu sp. n.

http://zoobank.org/46E6D8B1-FBE0-4226-9632-0B0D829FCD84

**Figure 1.**
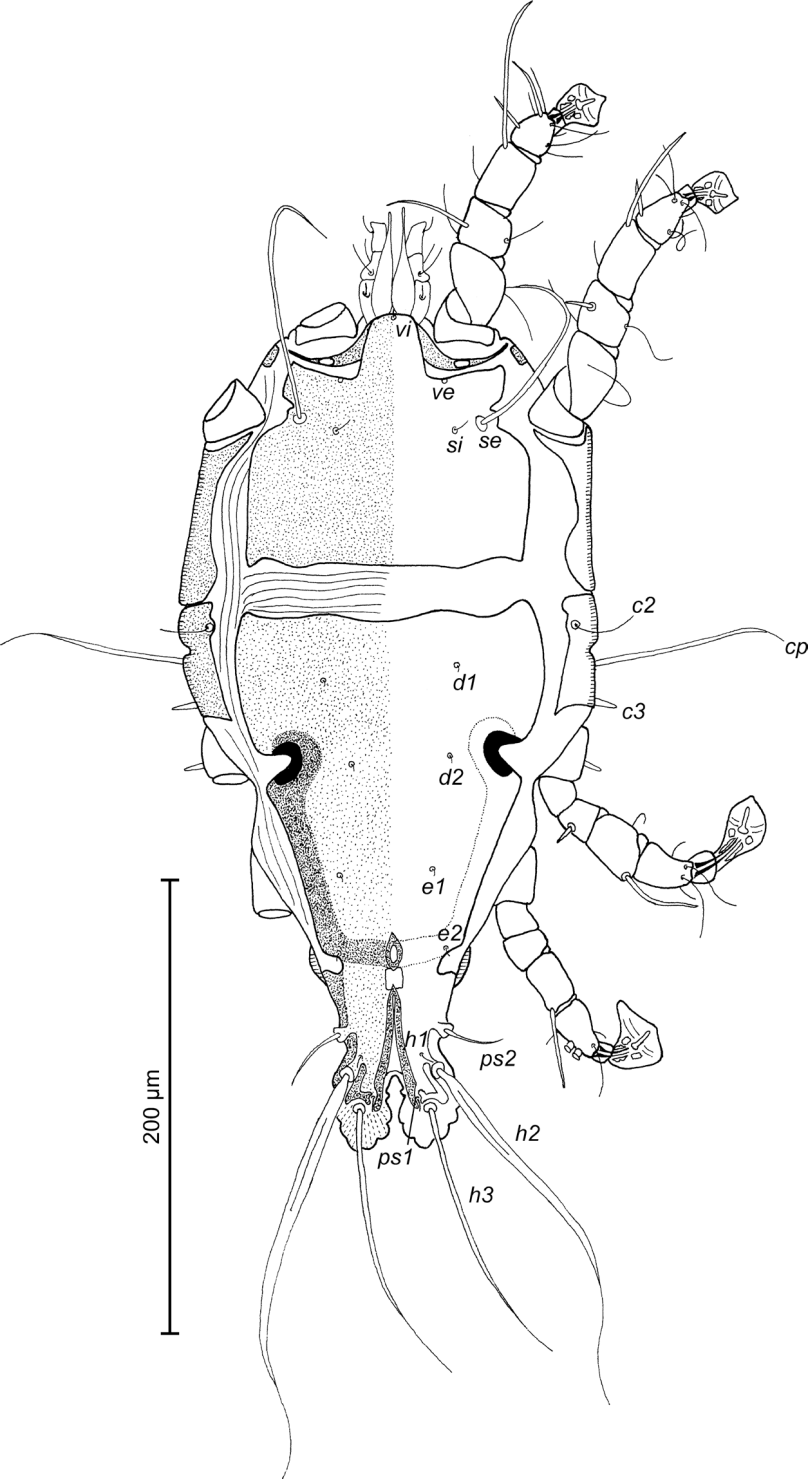
*Trouessartia cyanouropterae* sp. n., male holotype: dorsal view of idiosoma.

**Figure 2.**
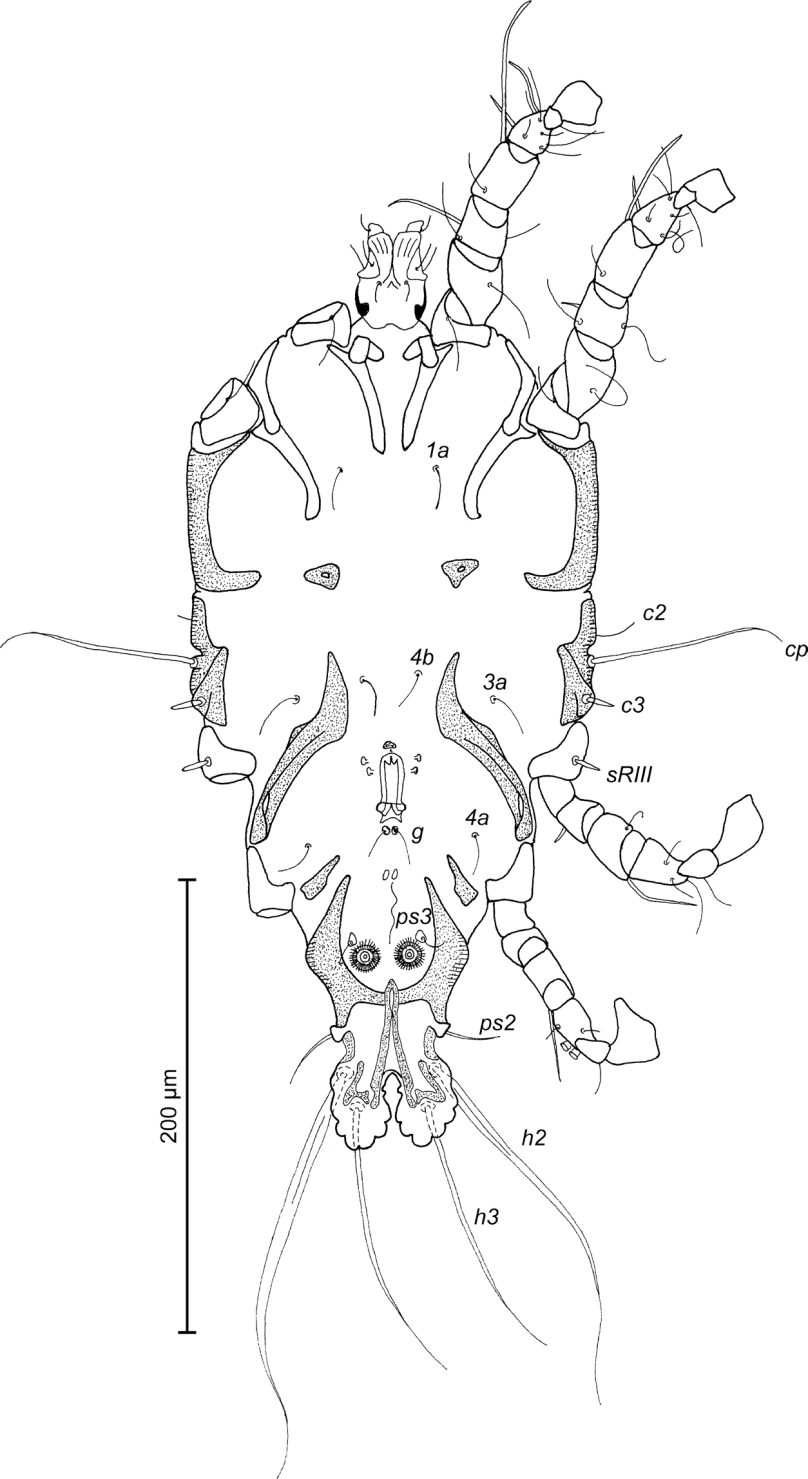
*Trouessartia cyanouropterae* sp. n., male holotype: ventral view of idiosoma.

**Figure 3.**
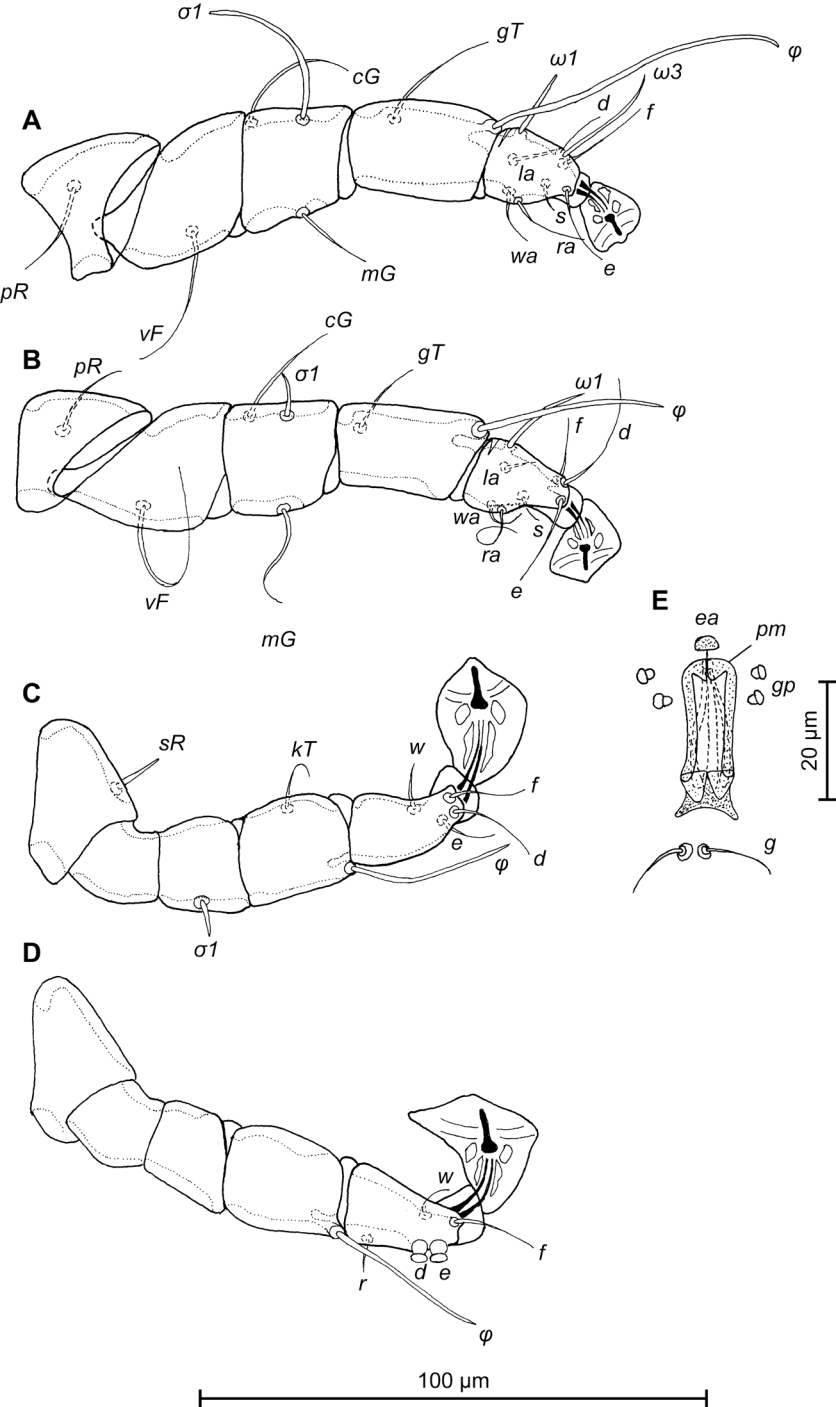
*Trouessartia cyanouropterae* sp. n., **A–D** details of male legs, dorsal view: **A** 9 leg I **B** leg II **C** leg III **D** leg IV **E** ventral view of male genital apparatus; Abbreviations: ea–epiandrum; gp–genital papillae; pm–parameres.

**Figure 4.**
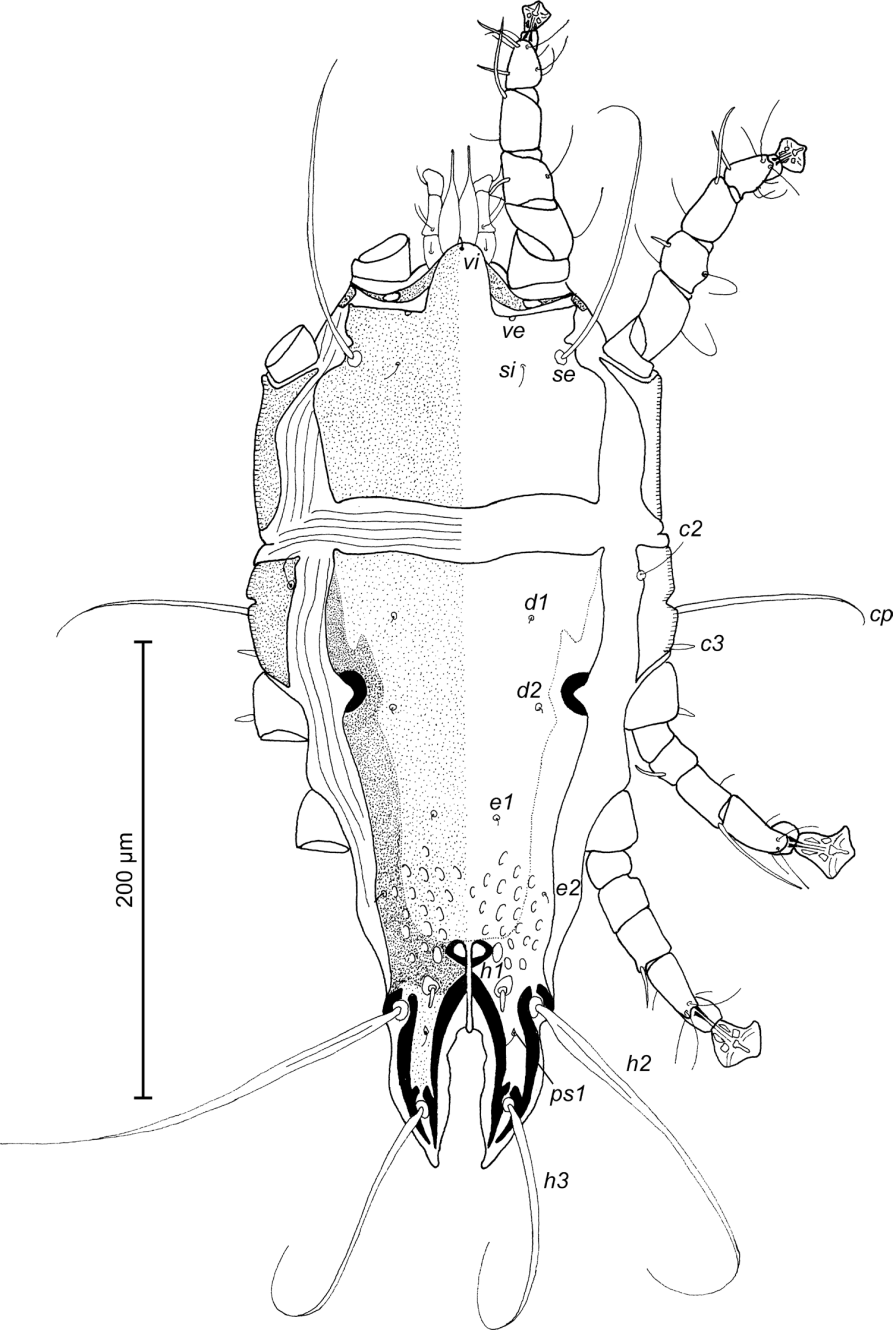
*Trouessartia cyanouropterae* sp. n., female paratype: dorsal view of idiosoma.

**Figure 5.**
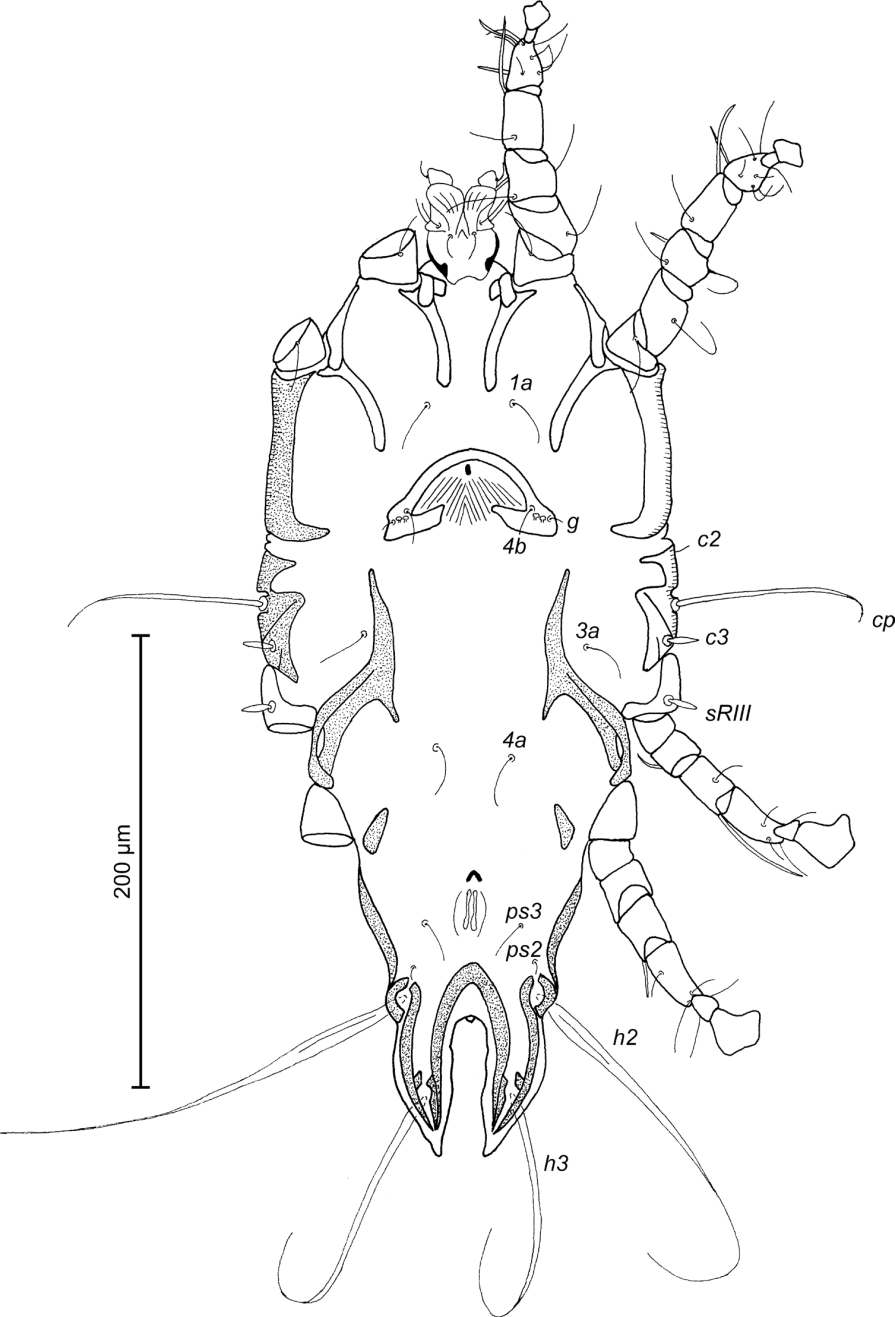
*Trouessartia cyanouropterae* sp. n., female paratype: ventral view of idiosoma.

**Figure 6.**
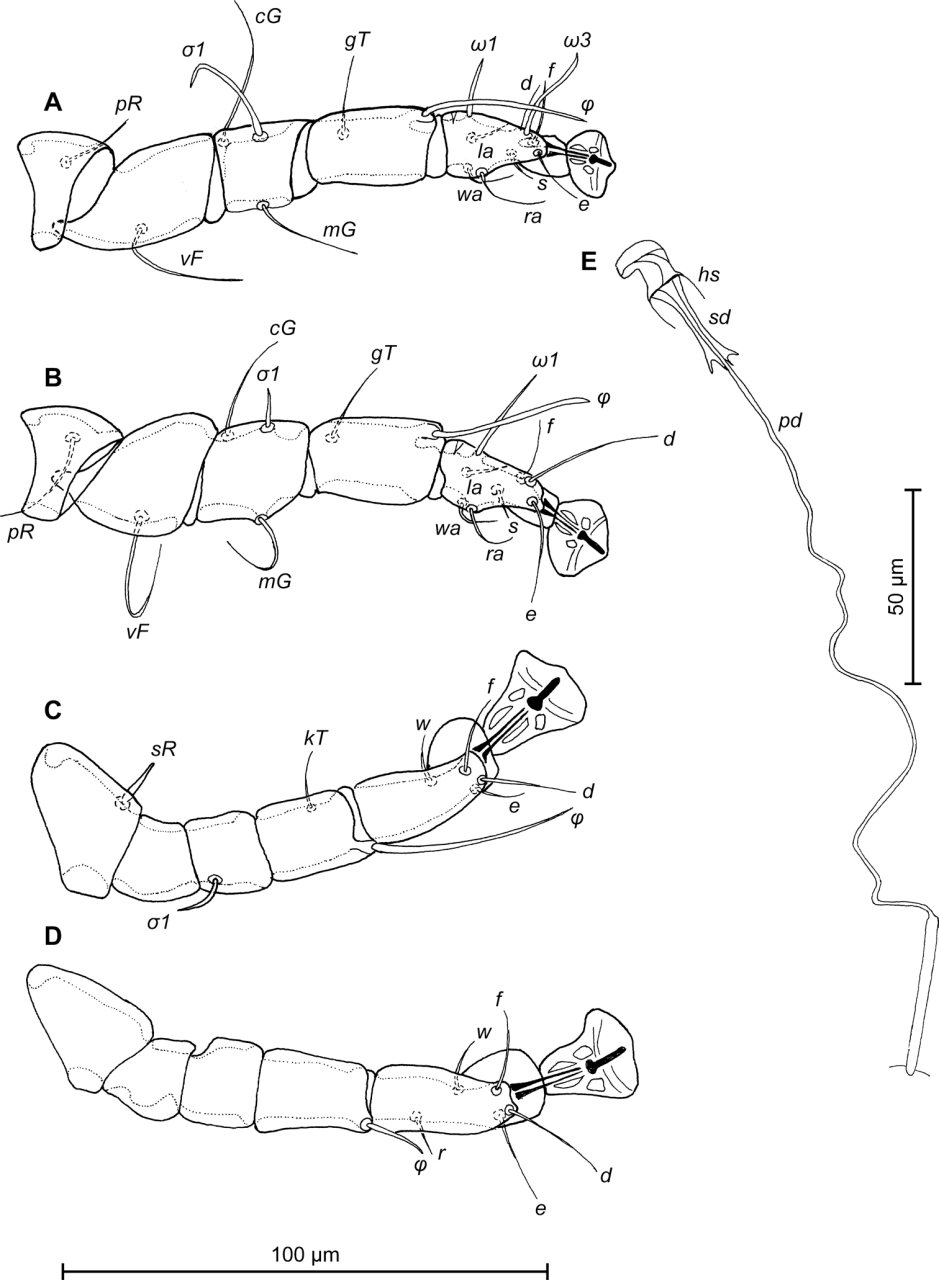
*Trouessartia cyanouropterae* sp. n., **A–D** details of female legs, dorsal view: **A** leg I **B** leg II **C** leg III **D** leg IV **E** spermatheca of female; Abbreviations: hs–head of spermatheca; pd–primary spermaduct; sd–secondary spermaduct.

Figs 1 , 2 , 3 , 4 , 5 , 6

##### Type material.

Male holotype (ANA623), 2 male (ANA624, ANA625) and 3 female (ANA626, ANA627, ANA628) paratypes 20.02.2013, from Blue-winged Minla *Actinodura cyanouroptera* (Hodgson) (Passeriformes, Leiothrichidae); **INDIA**: Meghalaya, Jaintia Hills, Khahnar village, (25°21'57.30"N, 92°36'51.72"E); 954 m; subtropical forest; collector D. Khlur B. Mukhim.

##### Description.

MALE (Figs 1; 2; 3A–E; holotype, range for 2 paratypes in parentheses): Length of idiosoma from anterior end to bases of setae *h3* 344 (331–332), greatest width at level of humeral shields 164 (164–165). Length of hysterosoma from sejugal furrow to bases of setae *h3* 224 (208–216). Prodorsal shield length along midline 110 (90–95), greatest width in posterior part 122 (118–119), lateral margins not fused with scapular shields, with antero-lateral extensions produced laterally between bases of legs I, II, surface without ornamentation (Fig. 1). Internal scapular setae *si* filiform, 10 (11–12) long, separated by 50 (48–54); external scapular setae *se* situated on prodorsal shield, 96 (102–106) long, separated by 82 (80–84). Vertical setae *ve* represented only by alveoli. Humeral shield with setae *c2* filiform, 22 (21–22) long. Setae *c3* narrowly lanceolate with acute apex, 12 (13–14) long. Dorsal hysterosoma with prohysteronotal shield and lobar shield connected, delimited from each other by lateral incisions immediately posterior to setae *e2* and small desclerotized median area of rectangular form. Prohysteronotal shield length 142 (130–140), width at anterior margin 118 (104–114), lateral margins incised at level of trochanters III, bottom of these incisions with C-shaped dark sclerotisation, dorsal hysterosomal apertures (DHA) absent. Dorsal setae *d1*, *d2* present, minute. Length of lobar shield excluding lamellae 66 (60–70). Apical parts of opisthosomal lobes approximate, separated by narrow terminal cleft, length of this cleft from anterior end to apices of lamellae 30 (28–30), width in anterior part 8 (8–9). Lamellae ovate in general shape, their margins with 5–6 rounded denticles, length from bases of setae *h3* to lamellar apices 16 (15–16). Setae *h1* anterior to setae *h2*. Distance between dorsal setae: *c2*-*d2* 68 (52–66), *d2*-*e2* 83 (74–79), *e2*-*h2* 50 (50–51), *h2*-*h3* 16 (16–17), *h2*-*h2* 34 (32–36), *h3*-*h3* 28 (27–30), *d1*-*d2* 39 (34–35), *e1*-*e2* 35 (31–34). Epimerites I free. Rudimentary sclerites rEpIIa present, roughly triangular. Genital apparatus situated between levels of trochanters III and IV, length 31 (28–29), greatest width 10 (9–10) (Fig. 2). Epiandrum present, small, setae *g* long and thin, almost touching at bases. Anterior genital papillae more distant from midline than posterior ones, postgenital plaque absent. Adanal apodemes heavily sclerotized, with narrow lateral membrane, without apophyses. Translobar apodeme present. Adanal shields small, triangular, bearing setae *ps3*. Anal suckers 11 (10–11) in diameter. Anterior ends of epimerites IV exceeding level of setae *4b*, epimerites IVa present, wide, anterior ends not reaching level of setae *4a.* Setae *4b* situated slightly anterior to level of setae *3a*, setae *g* and *4a* situated approximately at same transverse level. Distance between ventral setae: *4b-3a* 34 (33–34), *4b-g* 67 (62–64), *g-ps3* 46 (44–46), *ps3-h3* 72 (66–68). Setae *sR* of trochanters III short, narrowly lanceolate, with acute apex 10 (10–12) long. Tarsus IV 28 (24–25) long, modified setae *d* and *e* barrel-shaped, each with discoid cap, situated subapically (Fig. 3D). Legs IV with ambulacral disc extending to level of setae *h3*.

FEMALE (Figs 4; 5; 6A–E; range for 3 paratypes): Length of idiosoma from anterior end to apices of lamellar lobar processes 380–388, greatest width 170–176. Length of hysterosoma from sejugal furrow to apices of lamellar lobar processes 260–261. Prodorsal shield shaped as in male, 94–102 in length, 120–130 in width, surface without ornamentation. Setae *si* thin, filiform, 8–11 long, separated by 51–53, external scapular setae *se* situated on prodorsal shield, 104–108 long, separated by 70–86. Humeral shields with setae *c2* filiform, 22–23 long. Setae *c3* narrowly lanceolate, with acute apex, 10–12 in length. Hysteronotal shield length from anterior margin to bases of setae *h3* 228–232, width at anterior margin 112–116, lateral margins deeply incised at level of trochanters III, bottom of these incisions with C-shaped dark sclerotisation, DHA absent, posterior part with small ovate lacunae (Fig. 4). Dorsal setae *d1* present. Setae *h1* narrowly lanceolate with blunt apices, surrounded by triangular area of unsclerotized tegument, 8–10 long, situated antero-mesal to bases of setae *h2*, 17–22 from each lateral margin of hysteronotal shield. Setae *ps1* positioned dorsally on opisthosomal lobes, equidistant from outer and inner margins of lobe, closer to base of *h2* setae. Distance from bases of setae *h3* to membranous apices of lobes 24–26. Setae *f2* absent. Supranal concavity closed. Terminal cleft nearly parallel-sided, with tapering anterior end, length 71–74, width of cleft at level of setae *h3* 16–22. Interlobar membrane occupying anterior ¼ of terminal cleft, its anterior margin roughly rounded, lateral margins wavy; distance from its anterior margin to membranous lobar apices 54–58. External copulatory tube present, extremely short, 1–2 long, protruding from free margin of interlobar membrane. Spermatheca with primary spermaduct thickened in distal part, length of secondary spermaducts 25–30 (Fig. 6E). Distance between dorsal setae: *c2*-*d2* 59–71, *d2*-*e2* 80–94, *e2*-*h2* 42–46, *h2*-*h3* 38–42, *h2*-*h2* 56–60, *h3*-*h3* 36–42, *d1*-*d2* 30–41, *e1*-*e2* 40–42, *h1*-*h2* 14–16, *h1*-*h1* 29–34, *ps1*-*h3* 22–24. Epimerites I free. Epigynum 38–40 in length, 70–75 in width (Fig. 5). Epimerites IVa present, short. Setae *sR* of trochanters III narrowly lanceolate, with acute apex, 10–13 long. Legs IV with ambulacral disc extending to midlevel between setae *h2* and *h3*.

##### Etymology.

The name of the new species derives from the specific name of the type host and is a noun in the genitive case.

##### Remarks.

The new species *Trouessartia cyanouropterae* Constantinescu, sp. n. is most similar to *Trouessartia creatophorae* Mironov & Kopij, 1996, described from *Creatophora cinerea* (Meuschen) (Passeriformes, Sturnidae) in South Africa, (Mironov and Kopij 1996), in having, in both sexes, a similar shape of the hysteronotal shields with lateral margins deeply incised at the level of trochanters III, DHA absent, setae *d1* present, setae *c3* and *sRIII* narrow lanceolate and, in females, an ornamentation of ovoid lacunae in posterior part of the hysteronotal shield. Both sexes of *Trouessartia cyanuropterae* differ from *Trouessartia creatophorae* by the following features: setae *si* and *c2* are filiform (*vs.* setae *si* are narrow lanceolate, *c2* are long, needle-like in *Trouessartia creatophorae*). In males of *Trouessartia cyanouropterae* the margins of lamellae have 5-6 denticles, the rudimentary sclerites rEpIIa are roughly triangular, setae *g* are almost touching at bases, the genital apparatus is situated between levels of trochanters III and IV, setae *e* of tarsus IV is with discoid cap. In males of *Trouessartia creatophorae* the margins of lamellae have 9 denticles, the rudimentary sclerites rEpIIa are ovoid, setae *g* are separated, the genital apparatus is situated at level of trochanters IV, and seta *e* of tarsus IV is without a discoid cap. In females of the new species, the external copulatory tube is very short (1–2), setae *h1* are narrowly lanceolate, setae *ps1* are located closer to the base of *h2* setae then to *h3*. Females of *Trouessartia creatophorae* have a long external copulatory tube (about 19 long), setae *h1* are filiform, setae *ps1* are located closer to the base of *h3* setae.

#### Trouessartia alcippeae

Taxon classificationAnimaliaPasseriformesTrouessartiidae

Constantinescu sp. n.

http://zoobank.org/A49FF0FD-0D3C-4D9B-B7F3-A840E472F45E

**Figure 7.**
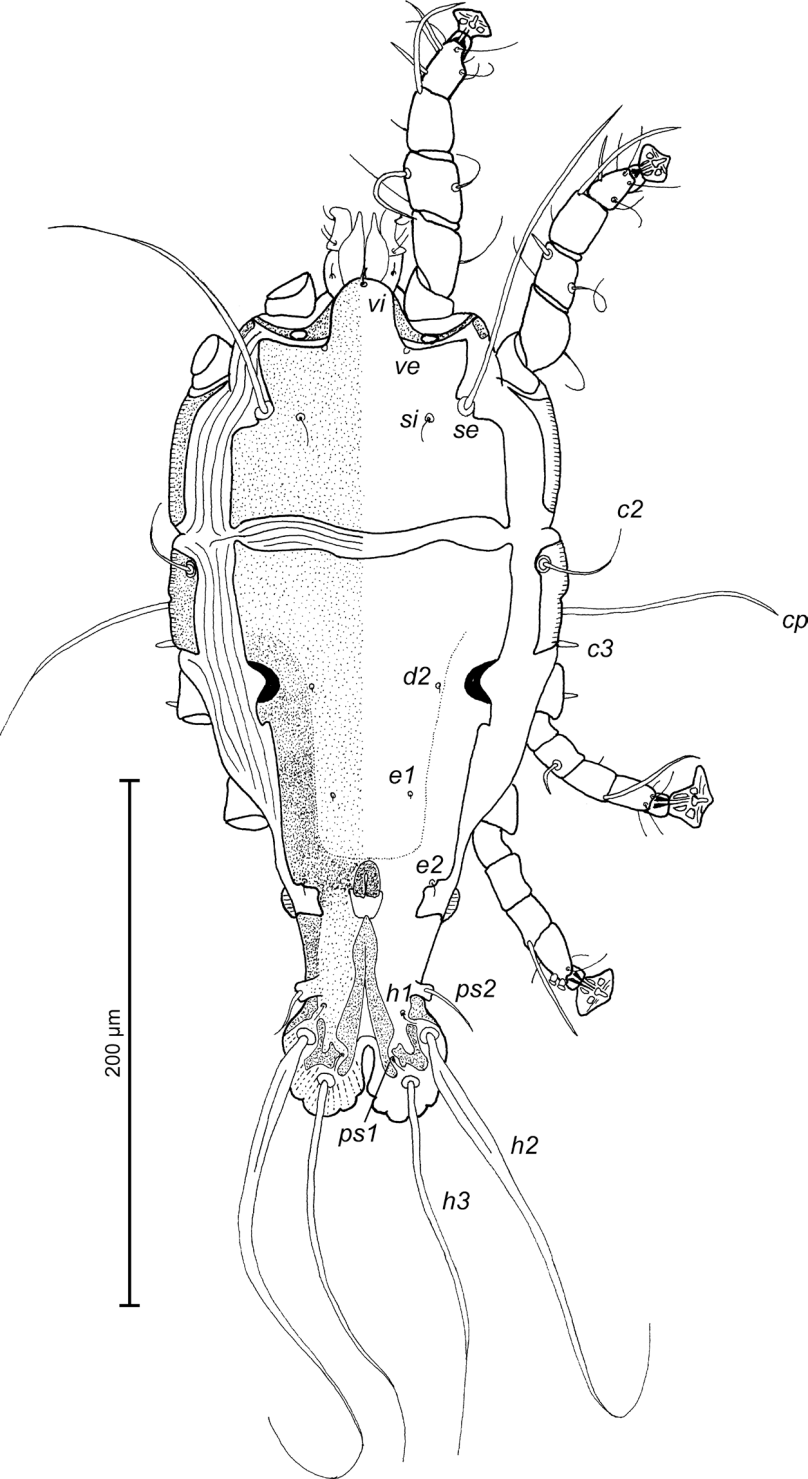
*Trouessartia alcippeae* sp. n., male holotype: dorsal view of idiosoma.

**Figure 8.**
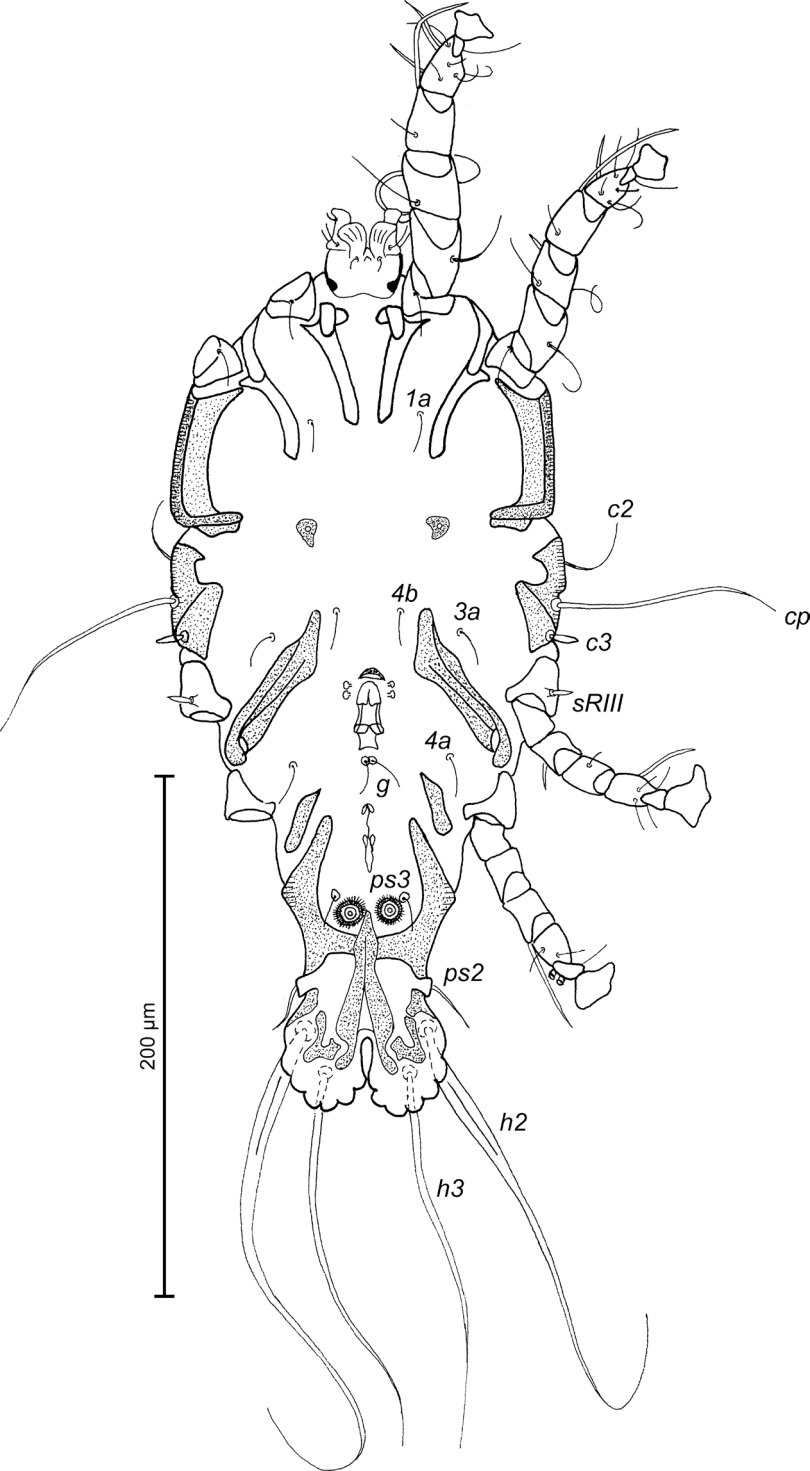
*Trouessartia alcippeae* sp. n., male holotype: ventral view of idiosoma.

**Figure 9.**
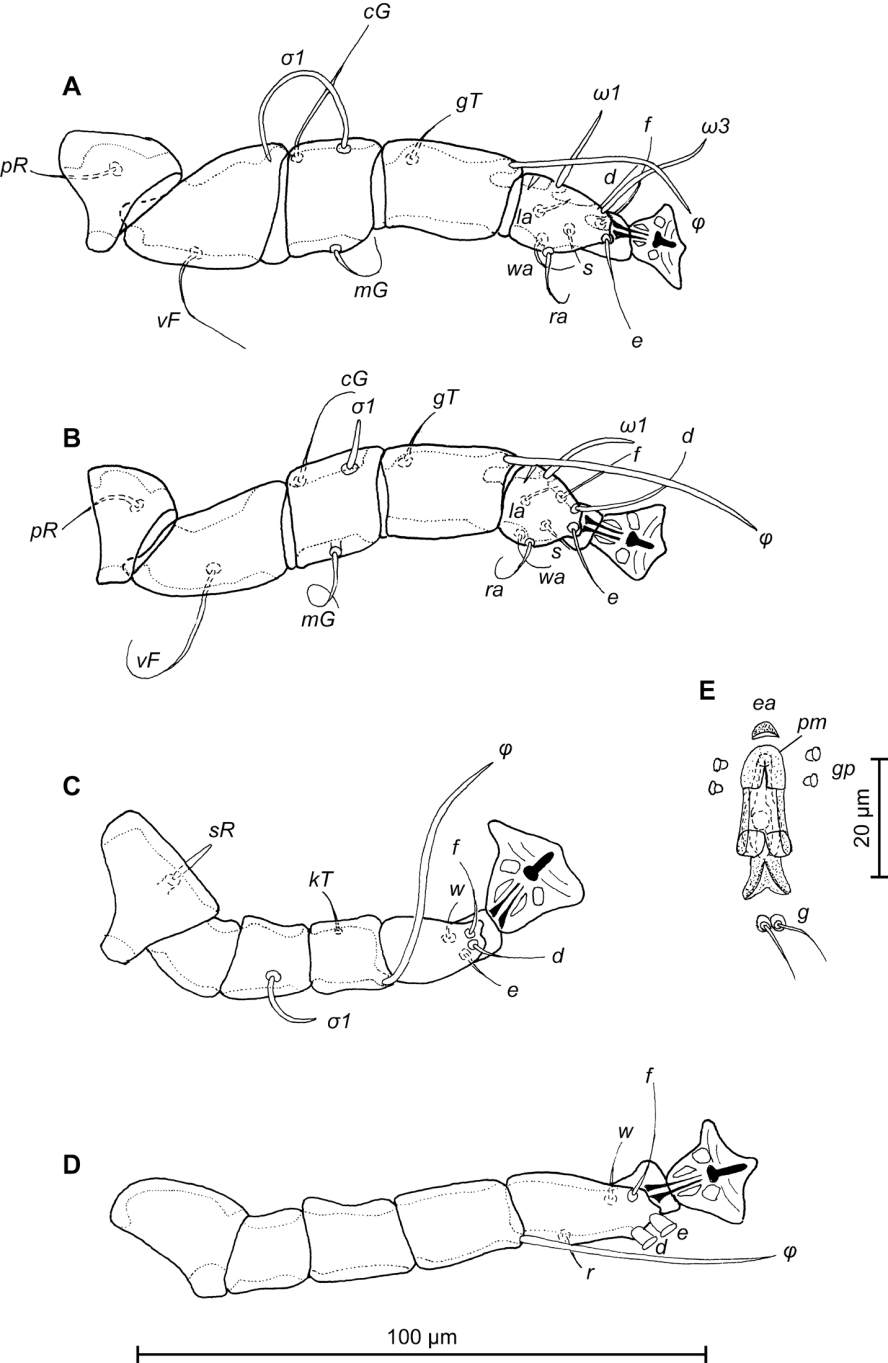
*Trouessartia alcippeae* sp. n., **A–D** details of male legs, dorsal view: **A** leg I **B** leg II **C** leg III **D** leg IV **E** ventral view of male genital apparatus; Abbreviations: ea–epiandrum; gp–genital papillae; pm–parameres.

**Figure 10.**
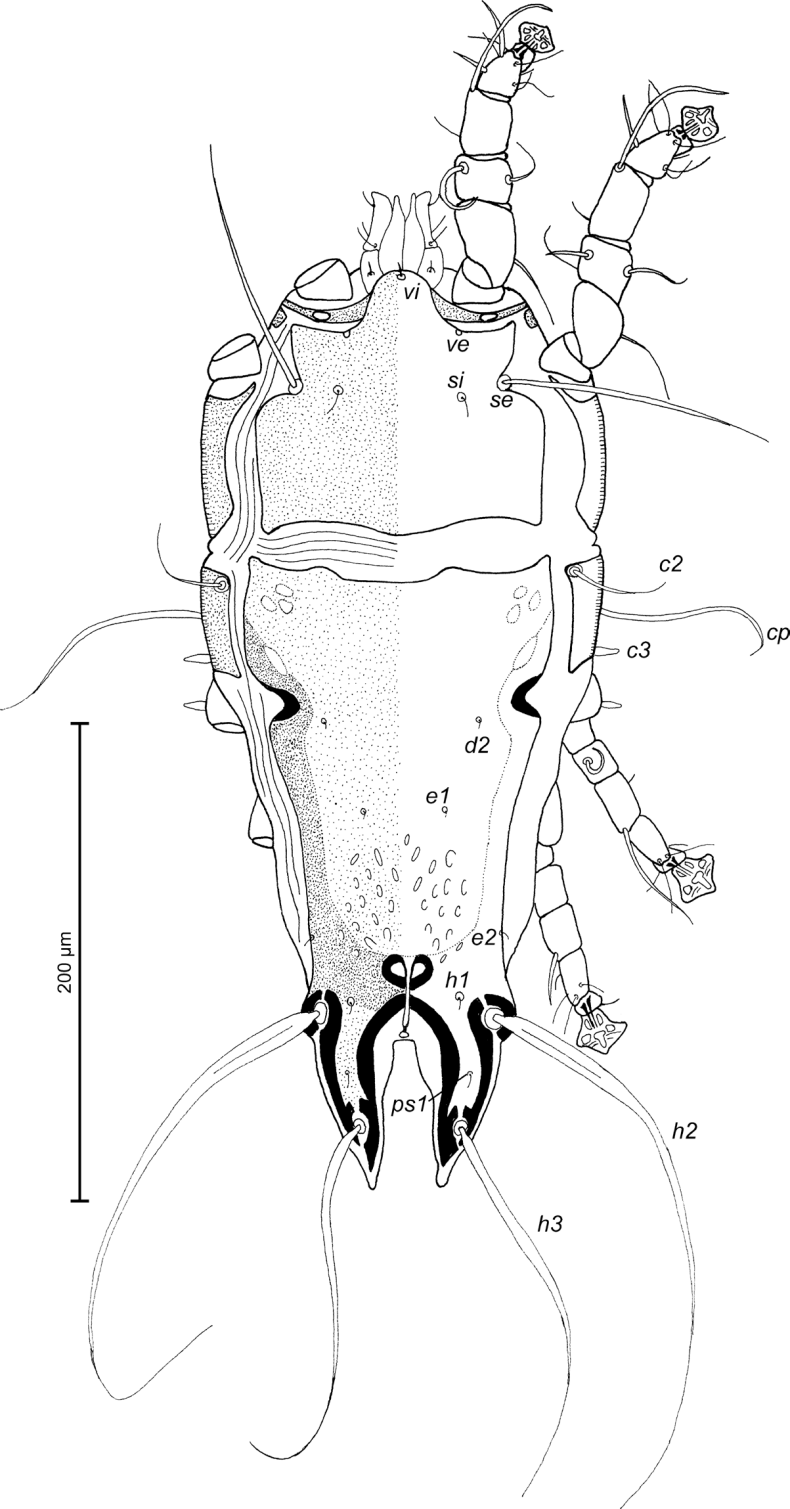
*Trouessartia alcippeae* sp. n., female paratype: dorsal view of idiosoma.

**Figure 11.**
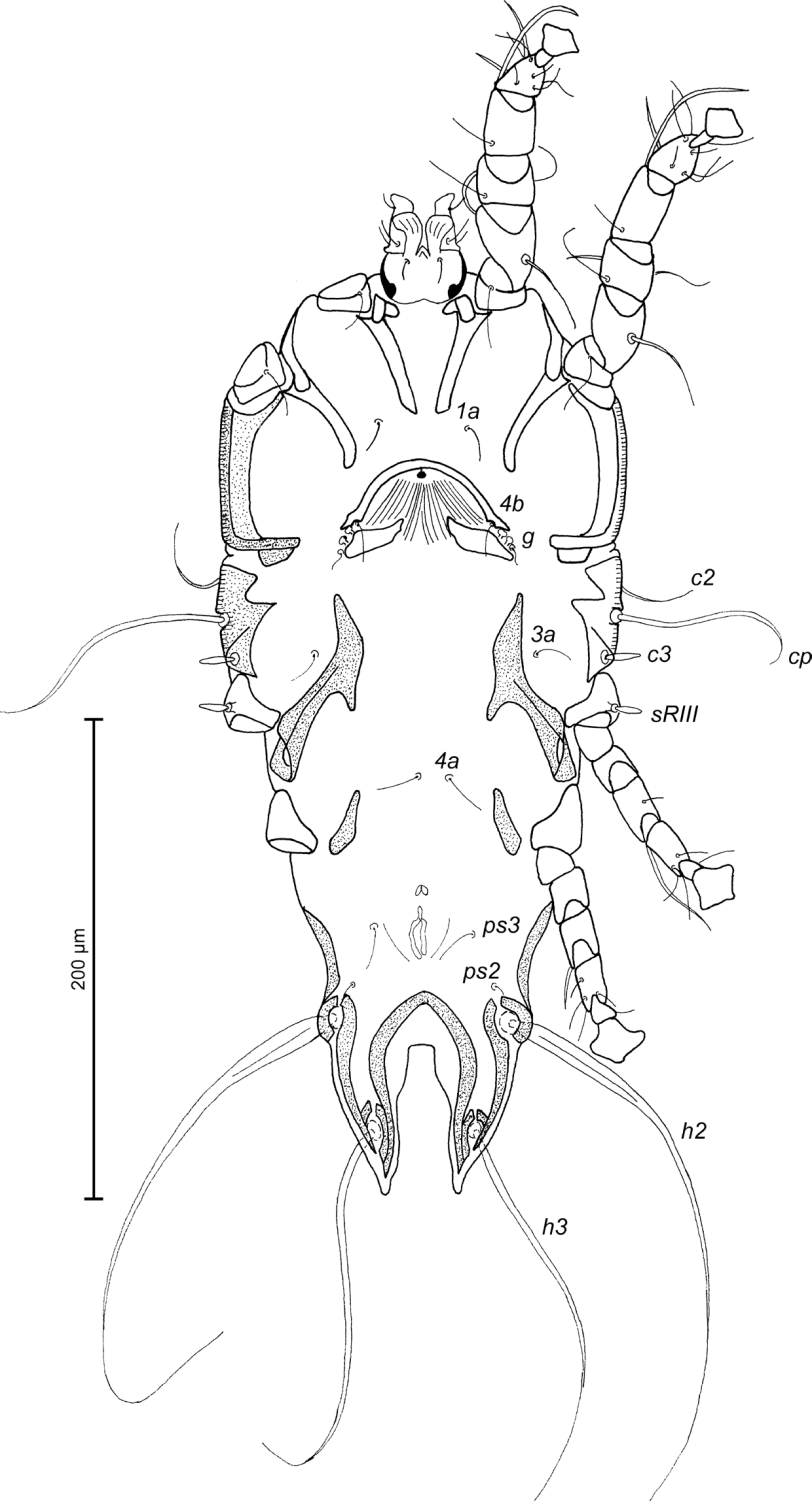
*Trouessartia alcippeae* sp. n., female paratype: ventral view of idiosoma.

**Figure 12.**
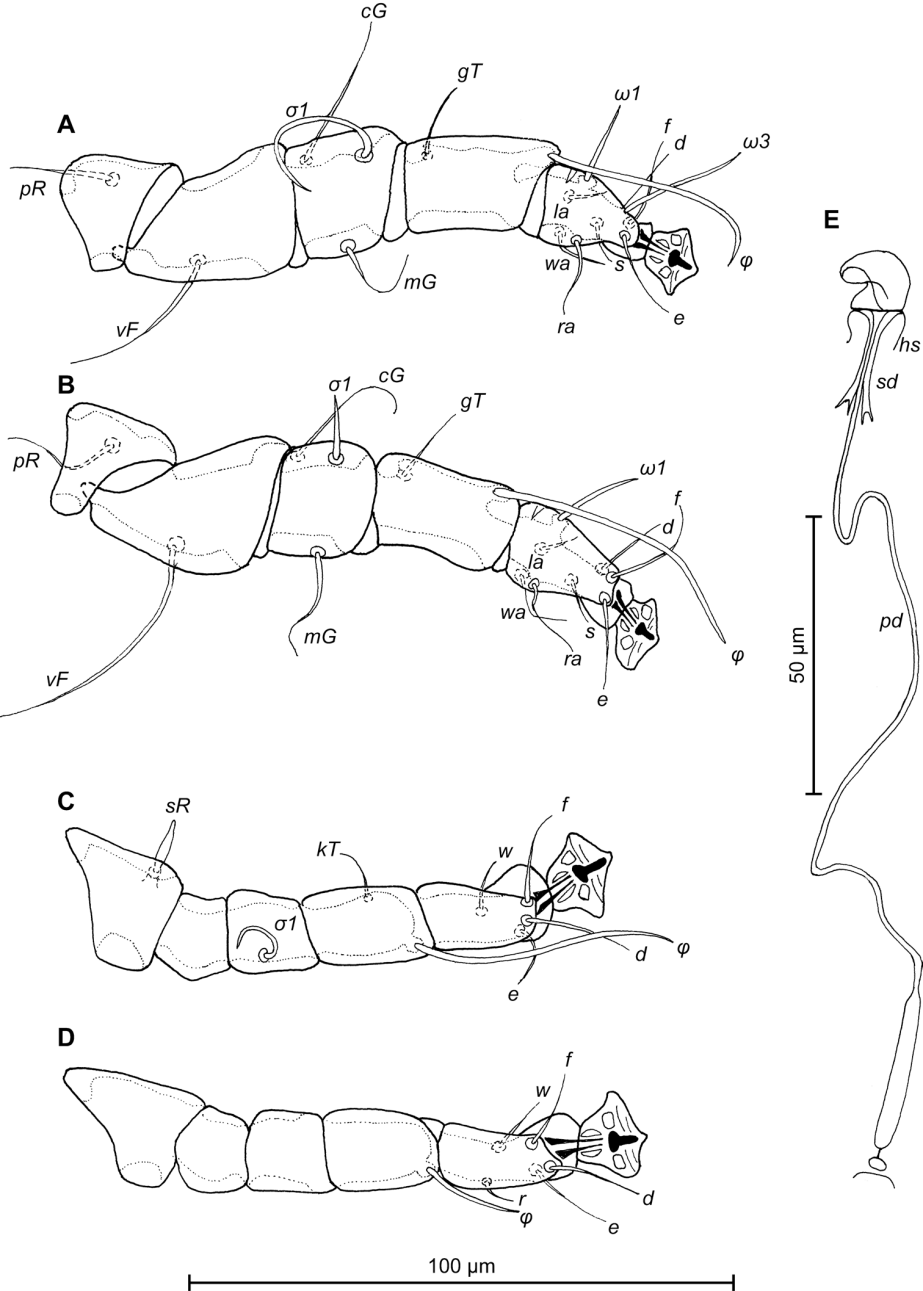
*Trouessartia alcippeae* sp. n., **A–D** details of female legs, dorsal view: **A** leg I **B** leg II **C** leg III **D** leg IV **E** spermatheca of female; Abbreviations: hs–head of spermatheca; pd–primary spermaduct; sd–secondary spermaduct.

Figs 7 , 8 , 9 , 10 , 11 , 12

##### Type material.

Male holotype (ANA639), 2 male (ANA641, ANA642) and 2 female (ANA640, ANA643) paratypes 10.02.2013, from Nepal Fulvetta *Alcippe nipalensis* (Hodgson) (Passeriformes, Leiothrichidae); **INDIA**: Meghalaya, Jaintia Hills, Khahnar village, (25°21'57.30"N, 92°36'51.72"E); 954 m; subtropical forest; collector D. Khlur B. Mukhim.

##### Description.

MALE (Figs 7; 8; 9A–E; holotype, range for 2 paratypes in parentheses): Length of idiosoma from anterior end to bases of setae *h3* 300 (304–324), greatest width at level of humeral shields 144 (152–154). Length of hysterosoma from sejugal furrow to bases of setae *h3* 200 (200–208). Prodorsal shield length along midline 94 (96–97), greatest width in posterior part 100 (108–110), lateral margins not fused with scapular shields, with antero-lateral extensions produced laterally between bases of legs I, II, surface without ornamentation (Fig. 7). Internal scapular setae *si* filiform, 7 (7–8) long, separated by 48 (50–52); external scapular setae *se* situated near lateral margins of prodorsal shield, 85 (94–106) long, separated by 72 (76–78). External vertical setae *ve* represented only by alveoli. Humeral shield with setae *c2* filiform, gradually tapering to apex 30 (30–40) long. Setae *c3* narrowly lanceolate, with acute apex, 11 (12–13) long. Dorsal hysterosoma with prohysteronotal and lobar shields connected, they delimited from each other by lateral incisions immediately posterior to setae *e2* and small unsclerotized medial area of trapezoidal form. Prohysteronotal shield length 128 (130–136), width at anterior margin 100 (96–108), lateral margins incised at level of trochanters III, dorsal hysterosomal apertures (DHA) absent. Dorsal setae *d1* absent, setae *d2* present, minute. Length of lobar shield excluding lamellae 56 (63–64). Apical parts of opisthosomal lobes approximate, separated by narrow terminal cleft, length of this cleft from anterior end to apices of lamellae 31 (29–30), width in anterior part 5 (5–7). Lamellae ovate in general shape, their margins with 4–7 rounded denticles, length from bases of setae *h3* to lamellar apices 16 (14–16). Setae *h1* anterior to setae *h2*. Distance between dorsal setae: *c2*-*d2* 62 (61–67), *d2*-*e2* 68 (74–76), *e2*-*h2* 50 (52–53), *h2*-*h3* 16 (16–17), *h2*-*h2* 38 (38–40), *h3*-*h3* 32 (31–32), *e1*-*e2* 32 (36–38). Epimerites I free. Rudimentary sclerites rEpIIa present, roughly triangular. Genital apparatus situated between levels of trochanters III and IV, length 30 (28–30), greatest width 9 (9–10) (Fig. 8). Epiandrum present, small, setae *g* long and thin, touching at bases, postgenital plaque absent. Anterior and posterior genital papillae at the same distance from midline. Adanal apodemes heavily sclerotized, with narrow lateral membrane, without apophyses. Translobar apodeme present. Adanal shields small, almost ovoid, bearing setae *ps3*. Anal suckers 10 (9–10) in diameter. Anterior ends of epimerites IV reaching level of setae *4b*, epimerites IVa present, wide, anterior ends not reaching level of setae *4a.* Setae *4b* situated slightly anterior to level of setae *3a*, setae *g* and *4a* situated approximately at same transverse level. Distance between ventral setae: *4b-3a* 27 (28–31), *4b-g* 56 (54–56), *g-ps3* 52 (52–54), *ps3-h3* 62 (62–64). Setae *sR* of trochanters III short, narrowly lanceolate, with acute apex, 13 (11–14) long. Tarsus IV 24 (24–26) long, modified setae *d* and *e* barrel-shaped, with discoid cap, situated subapically (Fig. 9D). Legs IV with ambulacral disc extending to level of setae *h2*.

FEMALE (Figs 10; 11; 12A–E; range for 2 paratypes): Length of idiosoma from anterior end to apices of lamellar lobar processes 376–377, greatest width 160–164. Length of hysterosoma from sejugal furrow to apices of lamellar lobar processes 252–260. Prodorsal shield shaped as in male, 102–106 in length, 112–118 in width, surface without ornamentation. Setae *si* thin, filiform, 10–11 long, separated by 55–57, external scapular setae *se* situated near lateral margins of prodorsal shield, 185–184 long, separated by 83–87. Humeral shields with setae *c2* filiform, gradually tapering to apex 39–44 long. Setae *c3* narrowly lanceolate, with acute apex, 12–14 in length. Hysteronotal shield length from anterior margin to bases of setae *h3* 226–232, width at anterior margin 108–112, lateral margins deeply incised at level of trochanters III, these incision with heavy C-shaped sclerotization, DHA absent, posterior part with small ovate lacunae (Fig. 10). Dorsal setae *d1* absent. Setae *h1* filiform, 5–6 long, situated antero-mesal to bases of setae *h2*, 17–18 from each lateral margin of hysteronotal shield. Setae *ps1* positioned dorsally on opisthosomal lobes, equidistant from outer and inner margins of lobe, closer to base of *h3* setae. Distance from bases of setae *h3* to membranous apices of lobes 26–32. Setae *f2* absent. Supranal concavity closed. Terminal cleft as an inverted U, length 78–86, width of cleft at level of setae *h3* 25–26. Interlobar membrane occupying anterior ¼ of terminal cleft, distance from free margin of membrane to membranous lobar apices 60–70. External copulatory tube absent, copulatory opening dorsally on interlobar membrane. Spermatheca with primary spermaduct thickened at base, length of secondary spermaducts 19–20 (Fig. 12E). Distance between dorsal setae: *c2*-*d2* 63–66, *d2*-*e2* 76–86, *e2*-*h2* 32–46, *h2*-*h3* 42–44, *h2*-*h2* 64–66, *h3*-*h3* 40–42, *e1*-*e2* 50–60, *h1*-*h2* 16–18, *h1*-*h1* 40–46, *ps1*-*h3* 22–26. Epimerites I free. Epigynum 39–40 in length, 72–76 in width (Fig. 11). Epimerites IVa present, short. Setae *sR* of trochanters III narrowly lanceolate, 11–12 long. Legs IV extending by ambulacral disc to midlevel between setae *h2* and *ps1*.

##### Etymology.

The specific epithet derives from the generic name of the type host and is a noun in the genitive case.

##### Remarks.

The new species, *Trouessartia alcippeae* Constantinescu, sp. n., is very similar in appearance to *Trouessartia cyanouropterae* described above in having, in both sexes, the dorsal shields similar in shape, the hysteronotal (prohysteronotal in males) shield with the lateral margins deeply incised at the level of trochanters III, DHA absent, and setae *c3* and *sRIII* narrow lanceolate. Males of the both species have a similar shape of epimerites (except epimerites IV), the lamellae ovate with rounded denticles, the setae *g* are close to each other, and the setae *d* and *e* are barrel-shaped, with a discoid cap, and situated apically. Females of the both species have a similar ornamentation of hysteronotal shield (ovoid lacunae), and the spermatheca is similar in shape. Both sexes of *Trouessartia alcippeae* differ from *Trouessartia cyanouropterae*, by the following characters: the setae *d1* are absent and setae *se* are situated on the lateral margins of prodorsal shield. In *Trouessartia cyanouropterae*, setae *d1* are present and setae *se* are situated on the prodorsal shield. Males of *Trouessartia alcippeae* have a small unsclerotized median area of trapezoidal form between the prohysteronotal shield and the lobar shield, epimerites IV are shorter and reach the level of setae *4b*, and the anterior and posterior genital papillae are at the same distance from midline. Males of *Trouessartia cyanouropterae* have a small rectangular unsclerotized area between the prohysteronotal shield and the lobar shield, epimerites IV are longer and exceeding the level of setae *4b*, and the anterior genital papillae are more distant from the midline than the posterior ones. Females of *Trouessartia alcippeae* have the setae *h1* filiform, the setae *ps1* are located closer to bases of setae *h3* and the the external copulatory tube is absent. Females of *Trouessartia cyanouropterae* have the setae *h1* lanceolate, the setae *ps1* are located closer to the base of *h2* setae and the external copulatory tube is present.

## Supplementary Material

XML Treatment for Trouessartia cyanouropterae

XML Treatment for Trouessartia alcippeae
